# Supplementary material for: An age-structured mathematical model of tetanus epidemiology, immunity, and vaccination across the life course
Source: Front Public Health. 2026 Oct 12;14:1903617. doi: 10.3389/fpubh.2026.1903617 (PMC13696406; doi:10.3389/fpubh.2026.1903617)
Supplement: Supplementary file 1 [file Supplementary_file_1.pdf]

# *Supplementary Material*

An age-structured mathematical model of tetanus epidemiology, immunity, and vaccination across the life course

Authors: Rachel A. Hounsell[a,b], Jared Norman[b], Sheetal P. Silal[a,b]

[a] Centre for Global Health Research, Nuffield Department of Medicine, University of Oxford, Oxford, United Kingdom

[b] Modelling and Simulation Hub, Africa (MASHA), Department of Statistical Sciences, University of Cape Town, Cape Town, South Africa

**1. Age groups**

Table S.1. Model age groups

| Category | Band size | Age groups | |
|---|---|---|---|
| <6 months of age | Weeks | 0-5wks<br>6-7wks<br>8-9wks<br>10-11wks<br>12-13wks | 14-15wks<br>16-17wks<br>18-19wks<br>20-21wks<br>22-23wks<br>24-25wks |
| 6–23 months | Months | 6mths<br>7mths<br>8mths<br>9mths | 15mths<br>16mths<br>17mths<br>18mths |

| | | | |
|---|---|---|---|
| | | 10mths<br>11mths<br>12mths<br>13mths<br>14mths | 19mths<br>20mths<br>21mths<br>22mths<br>23mths |
| 2–15 years | Years | 2yrs<br>3yrs<br>4yrs<br>5yrs<br>6yrs<br>7yrs<br>8yrs | 9yrs<br>10yrs<br>11yrs<br>12yrs<br>13yrs<br>14yrs<br>15yrs |
| >15 years | 5-years | 16-19yrs<br>20-24yrs<br>25-29yrs<br>30-34yrs<br>35-39yrs<br>40-44yrs | 45-49yrs<br>50-54yrs<br>55-59yrs<br>60-64yrs<br>65-69yrs<br>70-74yrs<br>75+ |

## 2. Model structure

**Table S.2.** Tetanus model compartments (states), descriptions and key assumptions

| State | | Description | |
|---|---|---|---|
| S | S | Completely susceptible | Susceptible state that the population enters when born, if the mother was not vaccinated during pregnancy or was vaccinated but the infant is not protected (based on vaccine effectiveness).<br><br>Upon vaccination with the first dose of the primary series, the cohort moves into $V_{1\underline{P}}$ as a single dose is not assumed to be sufficient to mount a protective response against tetanus infection.<br><br>Upon infection, those infected can enter $I_S$, the corresponding infection state. |
| V | $V_{MI\_P}$ | Maternal vaccination, protected | State that the population enters when born, if the mother received a vaccine during pregnancy.<br><br>Direct maternal protection is conferred to the infant, who is not susceptible to infection in this state.<br><br>The protected/not-protected split is based on the effectiveness of maternal vaccination for the protection of the infant against infection.<br><br>Upon vaccination with the first dose of the primary series, the cohort moves into $V_{1P}$ or $V_{1\underline{P}}$ depending on vaccine effectiveness. |

| | | |
|---|---|---|
| | | Over time, immunity wanes allowing those to transition to S, where they become fully susceptible to infection.<br><br>We assume that there is no immune blunting from maternal vaccination. |
| $V_{1P}$, $V_{2P}$, $V_{3P}$, $V_{4P}$, $V_{5P}$, $V_{6P}$ | Tetanus vaccination, protected | State that the cohort enters when they receive each sequential DTPCV dose (dose 1 = $V_1$, dose 2 = $V_2$, dose 3 = $V_3$, dose 4 = $V_4$, dose 5 = $V_5$, dose 6 = $V_6$) and are either fully protected by the vaccination ($V_{1P}$, $V_{2P}$, $V_{3P}$, $V_{4P}$, $V_{5P}$, $V_{6P}$) or unprotected ($V_{1\underline{P}}$, $V_{2\underline{P}}$, $V_{3\underline{P}}$, $V_{4\underline{P}}$, $V_{5\underline{P}}$, $V_{6\underline{P}}$). |
| $V_{1\underline{P}}$, $V_{2\underline{P}}$, $V_{3\underline{P}}$ , $V_{4\underline{P}}$, $V_{5\underline{P}}$, $V_{6\underline{P}}$, | Tetanus vaccination, unprotected | The protected/not-protected split is based on the effectiveness of each subsequent dose for protection against infection.<br><br>Upon vaccination with the primary series, the cohort moves into $V_{jP}$ or $V_{j\underline{P}}$ depending on vaccine effectiveness, and so on for doses $j$ ~ 1-6.<br><br>We assume that those moving from a protected vaccination state to a higher dose state will remain fully protected by the vaccination (i.e. $V_{1P}$ will only ever move into $V_{2P}$, not $V_{2\underline{P}}$).<br><br>We assume that those moving from an unprotected vaccination state to a higher dose state can either remain unprotected or move into a protected state (i.e. $V_{1\underline{P}}$ can move into either $V_{2P}$ or $V_{2\underline{P}}$ depending on vaccine effectiveness).<br><br>We assume that all vaccine doses are cumulative, such that the eligible cohort would only receive higher doses in the schedule if they have received the previous doses (i.e. an individual can only move into $V_2$ if they move from $V_1$ not S). |

| | | | |
|---|---|---|---|
| | | | Over time, immunity wanes (at a dose specific rate) allowing a transition to the corresponding $V_{j\underline{P}}$ compartment (vaccinated, not protected).<br><br>Unprotected vaccination states can get infected. Upon infection, those infected enter the corresponding I compartment. |
| | $V_{MM1P}$, $V_{MM2P}$, $V_{MM3P}$ | Maternal vaccination, protected (1=lowest, 2=mid, 3=highest immunity level) | Upon maternal vaccination, pregnant women move into $V_{MM1-3}$ depending on their prior state of immunity. |
| | $V_{MM1\underline{P}}$, $V_{MM2\underline{P}}$, $V_{MM3\underline{P}}$ | Maternal vaccination, unprotected (1=lowest, 2=mid, 3=highest immunity level) | Infants born to mothers in protected $V_{MM}$ states will enter the model in $V_{MI}$.<br><br>After the duration of pregnancy, the population transitions to the corresponding M state (i.e. from $V_{MM2}$ to $M_1$). This captures the history of maternal vaccination and ongoing protection from maternal vaccination antibodies. |
| | $M_1$, $M_2$, $M_3$ | History of maternal vaccination, protected (1=lowest, 2=mid, 3=highest immunity level) | Over time, immunity from maternal vaccination wanes, allowing the population to transition to W, which is a waned state of partially protective immunity/greater susceptibility to symptomatic infection. |
| | $M_{1\underline{P}}$, $M_{2\underline{P}}$, $M_{3\underline{P}}$ | History of maternal vaccination, unprotected (1=lowest, 2=mid, 3=highest immunity level) | |
| I | $I_k$ | Infected (symptomatic) | Infection does not confer immunity. Therefore, upon recovery the infected population transitions back to the state they were in prior to infection $I_k$ $\forall$ k $\in$ $\{S, M_{1-3}, VM_{1-3}, V_{1-6}\}$ |

| | | | |
|---|---|---|---|
| | | | Population in these states are infected only, not infectious.<br>The infected states are distinct from each other to preserve immune history in the model.<br>All infected compartments are the same in terms of severity and infectiousness. |
| D | D | Tetanus-related mortality | Those infected with tetanus who die transition to D, which is a terminal state in the model. |

**3. Model equations**

The rates of change of the population in each epidemiological state in the tetanus model are described by the following set of ordinary differential equations, for each age group $i = 56$:

$$\begin{aligned}\frac{dS_i}{dt} = {} & (1 - trans_{nT})(1 - mprotect)births + \gamma_T(1 - \mu_T)Is_i + \tau_{mi}Vmi_p - \lambda S_i \\ & - \alpha_i\, cov_1(1 - \mu_i)S_i - \alpha_i(1 - cov_1)(1 - \mu_i)S_i \\ & + \alpha_{i-1}(1 - cov_1)(1 - \mu_{i-1})S_{i-1} - mrate_1 S_i - mrate_2\varepsilon_2 S_i \\ & - mrate_2(1 - \varepsilon_2)S_i - \mu_i S_i\end{aligned} \tag{S.1}$$

$$\begin{aligned}\frac{dIs_i}{dt} = {} & trans_{nT}(1 - mprotect)births + \lambda S_i - \gamma_T(1 - \mu_T)Is_i - \mu_T\gamma_T Is_i - \alpha_i(1 - \mu_i)Is_i \\ & + \alpha_{i-1}(1 - \mu_{i-1})Is_{i-1} - \mu_i Is_i\end{aligned} \tag{S.2}$$

$$\begin{aligned}\frac{dIvm1_i}{dt} = {} & \lambda Vmm1_{np_i} - \gamma_T(1 - \mu_T)Ivm1_i - \mu_T\gamma_T Ivm1_i - \alpha_i(1 - \mu_i)Ivm1_i \\ & + \alpha_{i-1}(1 - \mu_{i-1})Ivm1_{i-1} - \mu_i Ivm1_i\end{aligned} \tag{S.3}$$

$$\begin{aligned}\frac{dIvm2_i}{dt} = {} & \lambda Vmm2_{np_i} - \gamma_T(1 - \mu_T)Ivm2_i - \mu_T\gamma_T Ivm2_i - \alpha_i(1 - \mu_i)Ivm2_i \\ & + \alpha_{i-1}(1 - \mu_{i-1})Ivm2_{i-1} - \mu_i Ivm2_i\end{aligned} \tag{S.4}$$

$$\begin{aligned}\frac{dIvm3_i}{dt} = {} & \lambda Vmm3_{np_i} - \gamma_T(1 - \mu_T)Ivm3_i - \mu_T\gamma_T Ivm3_i - \alpha_i(1 - \mu_i)Ivm3_i \\ & + \alpha_{i-1}(1 - \mu_{i-1})Ivm3_{i-1} - \mu_i Ivm3_i\end{aligned}$$

(S. 5 )

$$\frac{dIm1_i}{dt} = \lambda M1_{np_i} - \gamma_T(1-\mu_T)Im1_i - \mu_T\gamma_T Im1_i - \alpha_i(1-\mu_i)Im1_i + \alpha_{i-1}(1-\mu_{i-1})Im1_{i-1} - \mu_i Im1_i$$

(S.6 )

$$\frac{dIm2_i}{dt} = \lambda M2_{np_i} - \gamma_T(1-\mu_T)Im2_i - \mu_T\gamma_T Im2_i - \alpha_i(1-\mu_i)Im2_i + \alpha_{i-1}(1-\mu_{i-1})Im2_{i-1} - \mu_i Im2_i$$

(S.7 )

$$\frac{dIm3_i}{dt} = \lambda M3_{np_i} - \gamma_T(1-\mu_T)Im3_i - \mu_T\gamma_T Im3_i - \alpha_i(1-\mu_i)Im3_i + \alpha_{i-1}(1-\mu_{i-1})Im3_{i-1} - \mu_i Im3_i$$

(S.8 )

$$\frac{dIv1_i}{dt} = \lambda V1_{np_i} - \gamma_T(1-\mu_T)Iv1_i - \mu_T\gamma_T Iv1_i - \alpha_i(1-\mu_i)Iv1_i + \alpha_{i-1}(1-\mu_{i-1})Iv1_{i-1} - \mu_i Iv1_i$$

(S.9 )

$$\frac{dIv2_i}{dt} = \lambda V2_{np_i} - \gamma_T(1-\mu_T)Iv2_i - \mu_T\gamma_T Iv2_i - \alpha_i(1-\mu_i)Iv2_i + \alpha_{i-1}(1-\mu_{i-1})Iv2_{i-1} - \mu_i Iv2_i$$

(S.10 )

$$\frac{dIv3_i}{dt} = \lambda V3_{np_i} - \gamma_T(1-\mu_T)Iv3_i - \mu_T\gamma_T Iv3_i - \alpha_i(1-\mu_i)Iv3_i + \alpha_{i-1}(1-\mu_{i-1})Iv3_{i-1} - \alpha_i\,\mu_i Iv3_i$$

(S.11 )

$$\frac{dIv4_i}{dt} = \lambda V4_{np_i} - \gamma_T(1-\mu_T)Iv4_i - \mu_T\gamma_T Iv4_i - \alpha_i(1-\mu_i)Iv4_i + \alpha_{i-1}(1-\mu_{i-1})Iv4_{i-1} - \alpha_i\,\mu_i Iv4_i$$

(S.12 )

$$\frac{dIv5_i}{dt} = \lambda V5_{np_i} - \gamma_T(1-\mu_T)Iv5_i - \mu_T\gamma_T Iv5_i - \alpha_i(1-\mu_i)Iv5_i + \alpha_{i-1}(1-\mu_{i-1})Iv5_{i-1} - \alpha_i\,\mu_i Iv5_i$$

(S.13 )

$$\frac{dIv6_i}{dt} = \lambda V6_{np_i} - \gamma_T(1-\mu_T)Iv6_i - \mu_T\gamma_T Iv6_i - \alpha_i(1-\mu_i)Iv6_i + \alpha_{i-1}(1-\mu_{i-1})Iv6_{i-1} - \mu_i Iv6_i$$

(S.14 )

$$\frac{dVmi_{p_i}}{dt} = (mprotect)births - \tau_{mi}Vmi_{p_i} - \alpha_i\, cov_1(1-\mu_i)Vmi_{p_i} - \alpha_i(1-cov_1)(1-\mu_i)Vmi_{p_i} + \alpha_{i-1}(1-cov_1)(1-\mu_{i-1})Vmi_{p_{i-1}} - \mu_i Vmi_{p_i} \tag{S.15}$$

$$\frac{dV1_{p_i}}{dt} = \alpha_i\, cov_1(1-\mu_i)Vmi_{p_i} - \tau_1 V1_{p_i} - \alpha_i\, cov_2(1-\mu_i)V1_{p_i} - \alpha_i(1-cov_2)(1-\mu_i)V1_{p_i} + \alpha_{i-1}(1-cov_2)(1-\mu_{i-1})V1_{p_{i-1}} - mrate_1 V1_{p_i} - mrate_2 V1_{p_i} - \mu_i V1_{p_i} \tag{S.16}$$

$$\frac{dV1_{np_i}}{dt} = \gamma_T(1-\mu_T)Iv1_i + \tau_1 V1_{p_i} + \alpha_i\, cov_1(1-\mu_i)S_i - \lambda V1_{np_i} - \alpha_i\, cov_2\, \varepsilon_2\,(1-\mu_i)V1_{np_i} - \alpha_i\, cov_2(1-\varepsilon_2)(1-\mu_i)V1_{np_i} - \alpha_i(1-cov_2)(1-\mu_i)V1_{np_i} + \alpha_{i-1}(1-cov_2)(1-\mu_{i-1})V1_{np_{i-1}} - mrate_1 V1_{np_i} - mrate_2\, \varepsilon_{m2}\, V1_{np_i} - mrate_2\,(1-\varepsilon_{m2})\, V1_{np_i} - \mu_i V1_{np_i} \tag{S.17}$$

$$\frac{dV2_{p_i}}{dt} = \alpha_i\, cov_2(1-\mu_i)V1_{p_i} + \alpha_i\, cov_2\, \varepsilon_2\,(1-\mu_i)V1_{np_i} - \alpha_i\, cov_3(1-\mu_i)V2_{p_i} - \tau_2 V2_{p_i} - \alpha_i(1-cov_3)(1-\mu_i)V2_{p_i} + \alpha_{i-1}(1-cov_3)(1-\mu_{i-1})V2_{p_{i-1}} - mrate_1 V2_{p_i} - mrate_2 V2_{p_i} - \mu_i V2_{p_i} \tag{S.18}$$

$$\frac{dV2_{np_i}}{dt} = \gamma_T(1-\mu_T)Iv2_i + \tau_2 V2_{p_i} + \alpha_i\, cov_2(1-\varepsilon_2)(1-\mu_i)V1_{np_i} - \lambda V2_{np_i} - \alpha_i\, cov_3\, \varepsilon_3\,(1-\mu_i)V2_{np_i} - \alpha_i\, cov_3(1-\varepsilon_3)(1-\mu_i)V2_{np_i} - \alpha_i(1-cov_3)(1-\mu_i)V2_{np_i} + \alpha_{i-1}(1-cov_3)(1-\mu_{i-1})V2_{np_{i-1}} - mrate_1 V2_{np_i} - mrate_2\, \varepsilon_{m2}\, V2_{np_i} - mrate_2\,(1-\varepsilon_{m2})\, V2_{np_i} - \mu_i V2_{np_i} \tag{S.19}$$

$$\frac{dV3_{p_i}}{dt} = \alpha_i\, cov_3(1-\mu_i)V2_{p_i} + \alpha_i\, cov_3\, \varepsilon_3\,(1-\mu_i)V2_{np_i} - \alpha_i\, cov_4(1-\mu_i)V3_{p_i} - \tau_3 V3_{p_i} - \alpha_i(1-cov_4)(1-\mu_i)V3_{p_i} + \alpha_{i-1}(1-cov_4)(1-\mu_{i-1})V3_{p_{i-1}} - mrate_1 V3_{p_i} - mrate_2 V3_{p_i} - \mu_i V3_{p_i} \tag{S.20}$$

$$\frac{dV3_{np_i}}{dt} = \gamma_T(1-\mu_T)Iv3_i + \tau_3 V3_{p_i} + \alpha_i\, cov_3(1-\varepsilon_3)(1-\mu_i)V2_{np_i} - \lambda V3_{np_i} - \alpha_i\, cov_4\, \varepsilon_4\,(1-\mu_i)V3_{np_i} - \alpha_i\, cov_4(1-\varepsilon_4)(1-\mu_i)V3_{np_i} - \alpha_i(1-cov_4)(1-\mu_i)V3_{np_i} + \alpha_{i-1}(1-cov_4)(1-\mu_{i-1})V3_{np_{i-1}} - mrate_1 V3_{np_i} - mrate_2\, \varepsilon_{m2}\, V3_{np_i} - mrate_2\,(1-\varepsilon_{m2})\, V3_{np_i} - \mu_i V3_{np_i}$$

(S.21)

$$\frac{dV4_{p_i}}{dt} = \alpha_i\, cov_4(1-\mu_i)V3_{p_i} + \alpha_i\, cov_4\, \varepsilon_4\, (1-\mu_i)V3_{np_i} - \alpha_i\, cov_5(1-\mu_i)V4_{p_i} - \tau_4 V4_{p_i} - \alpha_i(1-cov_5)(1-\mu_i)V4_{p_i} + \alpha_{i-1}(1-cov_5)(1-\mu_{i-1})V4_{p_{i-1}} - mrate_1 V4_{p_i} - mrate_2 V4_{p_i} - \mu_i V4_{p_i}$$

(S.22)

$$\frac{dV4_{np_i}}{dt} = \gamma_T(1-\mu_T)Iv4_i + \tau_4 V4_{p_i} + \alpha_i\, cov_4(1-\varepsilon_4)(1-\mu_i)V3_{np_i} - \lambda V4_{np_i} - \alpha_i\, cov_5\, \varepsilon_5\, (1-\mu_i)V4_{np_i} - \alpha_i\, cov_5(1-\varepsilon_5)(1-\mu_i)V4_{np_i} - \alpha_i(1-cov_5)(1-\mu_i)V4_{np_i} + \alpha_{i-1}(1-cov_5)(1-\mu_{i-1})V4_{np_{i-1}} - mrate_1 V4_{np_i} - mrate_2\, \varepsilon_{m2}\, V4_{np_i} - mrate_2\, (1-\varepsilon_{m2})\, V4_{np_i} - \mu_i V4_{np_i}$$

(S.23)

$$\frac{dV5_{p_i}}{dt} = \alpha_i\, cov_5(1-\mu_i)V4_{p_i} + \alpha_i\, cov_5\, \varepsilon_5\, (1-\mu_i)V4_{np_i} - \alpha_i\, cov_6(1-\mu_i)V5_{p_i} - \tau_5 V5_{p_i} - \alpha_i(1-cov_6)(1-\mu_i)V5_{p_i} + \alpha_{i-1}(1-cov_6)(1-\mu_{i-1})V5_{p_{i-1}} - mrate_1 V5_{p_i} - mrate_2 V5_{p_i} - \mu_i V5_{p_i}$$

(S.24)

$$\frac{dV5_{np_i}}{dt} = \gamma_T(1-\mu_T)Iv5_i + \tau_5 V5_{p_i} + \alpha_i\, cov_5(1-\varepsilon_5)(1-\mu_i)V4_{np_i} - \lambda V5_{np_i} - \alpha_i\, cov_6\, \varepsilon_6\, (1-\mu_i)V5_{np_i} - \alpha_i\, cov_6(1-\varepsilon_6)(1-\mu_i)V5_{np_i} - \alpha_i(1-cov_6)(1-\mu_i)V5_{np_i} + \alpha_{i-1}(1-cov_6)(1-\mu_{i-1})V5_{np_{i-1}} - mrate_1 V5_{np_i} - mrate_2\, \varepsilon_{m2}\, V5_{np_i} - mrate_2\, (1-\varepsilon_{m2})\, V5_{np_i} - \mu_i V5_{np_i}$$

(S.25)

$$\frac{dV6_{p_i}}{dt} = \alpha_i\, cov_{56}(1-\mu_i)V5_{p_i} + \alpha_i\, cov_6\, \varepsilon_6\, (1-\mu_i)V5_{np_i} - \tau_6 V6_{p_i} - \alpha_i(1-\mu_i)V5_{p_i} + \alpha_{i-1}(1-\mu_{i-1})V5_{p_{i-1}} - mrate_1 V6_{p_i} - mrate_2 V6_{p_i} - \mu_i V6_{p_i}$$

(S.26)

$$\frac{dV6_{np_i}}{dt} = \gamma_T(1-\mu_T)Iv6_i + \tau_6 V6_{p_i} + \alpha_i\, cov_6(1-\varepsilon_6)(1-\mu_i)V5_{np_i} - \lambda V6_{np_i} - \alpha_i(1-\mu_i)V6_{np_i} + \alpha_{i-1}(1-\mu_{i-1})V6_{np_{i-1}} - mrate_1 V6_{np_i} - mrate_2\, \varepsilon_{m2}\, V6_{np_i} - mrate_2\, (1-\varepsilon_{m2})\, V6_{np_i} - \mu_i V6_{np_i}$$

(S.27)

$$\frac{dM1_{p_i}}{dt} = \tau_{vmm} Vmm1_{p_i} - mrate_1 M1_{p_i} - mrate_2 M1_{p_i} - \tau_{mm} M1_{p_i} - \alpha_i(1-\mu_i)M1_{p_i} + \alpha_{i-1}(1-\mu_{i-1})M1_{p_{i-1}} - \mu_i M1_{p_i}$$

(S.28)

$$\frac{dM1_{np_i}}{dt} = \gamma_T(1-\mu_T)Im1_i + \tau_{mm}M1_{p_i} + \tau_{vmm}Vmm1_{np_i} - \lambda M1_{np_i} - mrate_1 M1_{np_i} - mrate_2\varepsilon_{m2}M1_{np_i} - mrate_2(1-\varepsilon_{m2})M1_{np_i} - \alpha_i(1-\mu_i)M1_{np_i} + \alpha_{i-1}(1-\mu_{i-1})M1_{np_{i-1}} - \mu_i M1_{np_i}$$

(S.29)

$$\frac{dM2_{p_i}}{dt} = \tau_{vmm}Vmm2_{p_i} - mrate_1 M2_{p_i} - mrate_2 M2_{p_i} - \tau_4 M2_{p_i} - \alpha_i(1-\mu_i)M2_{p_i} + \alpha_{i-1}(1-\mu_{i-1})M2_{p_{i-1}} - \mu_i M2_{p_i}$$

(S.30)

$$\frac{dM2_{np_i}}{dt} = \gamma_T(1-\mu_T)Im2_i + \tau_4 M2_{p_i} + \tau_{vmm}Vmm2_{np_i} - \lambda M2_{np_i} - mrate_1 M2_{np_i} - mrate_2\varepsilon_{m2}M2_{np_i} - mrate_2(1-\varepsilon_{m2})M2_{np_i} - \alpha_i(1-\mu_i)M2_{np_i} + \alpha_{i-1}(1-\mu_{i-1})M2_{np_{i-1}} - \mu_i M2_{np_i}$$

(S.31)

$$\frac{dM3_{p_i}}{dt} = \tau_{vmm}Vmm3_{p_i} - mrate_1 M3_{p_i} - mrate_2 M3_{p_i} - \tau_6 M3_{p_i} - \alpha_i(1-\mu_i)M3_{p_i} + \alpha_{i-1}(1-\mu_{i-1})M3_{p_{i-1}} - \mu_i M3_{p_i}$$

(S.32)

$$\frac{dM3_{np_i}}{dt} = \gamma_T(1-\mu_T)Im3_i + \tau_6 M3_{p_i} + \tau_{vmm}Vmm3_{np_i} - \lambda M3_{np_i} - mrate_1 M3_{np_i} - mrate_2\varepsilon_{m2}M3_{np_i} - mrate_2(1-\varepsilon_{m2})M3_{np_i} - \alpha_i(1-\mu_i)M3_{np_i} + \alpha_{i-1}(1-\mu_{i-1})M3_{np_{i-1}} - \mu_i M3_{np_i}$$

(S.33)

$$\frac{dVmm1_{p_i}}{dt} = mrate_2\varepsilon_2 S_i + mrate_1(V1_{p_i} + V2_{p_i}) + mrate_2 V1_{p_i} + mrate_2\,\varepsilon_{m2}\left(V1_{np_i} + V2_{np_i} + V3_{np_i} + V4_{np_i} + V5_{np_i} + V6_{np_i} + M1_{np_i} + M2_{np_i} + M3_{np_i}\right) - \tau_{vmm}Vmm1_{p_i} - \alpha_i(1-\mu_i)Vmm1_{p_i} + \alpha_{i-1}(1-\mu_{i-1})Vmm1_{p_{i-1}} - \mu_i Vmm1_{p_i}$$

(S.34)

$$\frac{dVmm1_{np_i}}{dt} = \gamma_T(1-\mu_T)Ivm1_i + mrate_1\left(S_i + V1_{np_i} + V2_{np_i} + V3_{np_i} + V4_{np_i} + V5_{np_i} + V6_{np_i} + M1_{np_i} + M2_{np_i} + M3_{np_i}\right) + mrate_2(1-\varepsilon_2)S_i + mrate_2(1-\varepsilon_{m2})\left(V1_{np_i} + V2_{np_i} + V3_{np_i} + V4_{np_i} + V5_{np_i} + V6_{np_i} + M1_{np_i} + M2_{np_i} + M3_{np_i}\right) - \tau_{vmm}Vmm1_{np_i} - \lambda Vmm1_{np_i} - \alpha_i(1-\mu_i)Vmm1_{np_i} + \alpha_{i-1}(1-\mu_{i-1})Vmm1_{np_{i-1}} - \mu_i Vmm1_{np_i}$$

*( S.35 )*

$$\frac{dVmm2_{p_i}}{dt} = mrate_1(V3_{p_i} + M1_{p_i}) + mrate_2(V2_{p_i} + M1_{p_i}) - \tau_{vmm}Vmm2_{p_i} - \alpha_i(1-\mu_i)Vmm2_{p_i} + \alpha_{i-1}(1-\mu_{i-1})Vmm2_{p_{i-1}} - \mu_i Vmm2_{p_i}$$

*( S.36 )*

$$\frac{dVmm2_{np_i}}{dt} = (1-\mu_T)\gamma_T Ivm2_i - \tau_{vmm}Vmm2_{np_i} - \lambda Vmm2_{np_i} - \alpha_i(1-\mu_i)Vmm2_{np_i} + \alpha_{i-1}(1-\mu_{i-1})Vmm2_{np_{i-1}} - \mu_i Vmm2_{np_i}$$

*( S.37 )*

$$\frac{dVmm3_{p_i}}{dt} = mrate_1(V4_{p_i} + V5_{p_i} + V6_{p_i} + M2_{p_i} + M3_{p_i}) + mrate_2(V3_{p_i} + V4_{p_i} + V5_{p_i} + V6_{p_i} + M2_{p_i} + M3_{p_i}) - \tau_{vmm}Vmm3_{p_i} - \alpha_i(1-\mu_i)Vmm3_{p_i} + \alpha_{i-1}(1-\mu_{i-1})Vmm3_{p_{i-1}} - \mu_i Vmm3_{p_i}$$

*( S.38 )*

$$\frac{dVmm3_{np_i}}{dt} = (1-\mu_T)\gamma_T Ivm3_i - \tau_{vmm}Vmm3_{np_i} - \lambda Vmm3_{np_i} - \alpha_i(1-\mu_i)Vmm3_{np_i} + \alpha_{i-1}(1-\mu_{i-1})Vmm3_{np_{i-1}} - \mu_i Vmm3_{np_i}$$

*( S.39 )*

$$\frac{dD_i}{dt} = \mu_T\,\gamma_T\,(Is_i + Iv1_i + Iv2_i + Iv3_i + Iv4_i + Iv5_i + Iv6_i + Ivm1_i + Im1_i + Ivm2_i + Im2_i + Ivm3_i + Im3_i)$$

*( S.40 )*

## 4. Parameters

Table S.3. Model parameters for tetanus

| Symbol | Parameter | Unit^ | Value | Range | Distribution* | Source |
|---|---|---|---|---|---|---|
| Disease-related parameters | | | | | | |
| $\gamma$ | Recovery rate | Days | 30 | [14, 60] | Triangular | (Roper et al., 2018) |
| $\mu_{T_t}$ | Tetanus mortality: Percentage of treated cases resulting in death | % | 50 | [25, 75] | Triangular | (Roper et al., 2018; World Health Organization, 2017) |
| $\mu_{T_{nt}}$ | Tetanus mortality: Percentage of untreated cases resulting in death | % | 75 | [50, 100] | Triangular | (Roper et al., 2018; World Health Organization, 2017) |
| λ | Force of infection = Transmission scalar (calibration parameter) | - | Model derived | | | |
| Vaccine-related parameters | | | | | | |
| $\tau_1$<br>$\tau_2$<br>$\tau_3$<br>$\tau_4$<br>$\tau_5$<br>$\tau_6$ | Immunity waning rate from vaccination (duration of protection against infection)<br>• $V_{1P}$ to $V_{1NP}$ (1 dose)<br>• $V_{2P}$ to $V_{2NP}$ (2 doses)<br>• $V_{3P}$ to $V_{3NP}$ (3 doses)<br>• $V_{4P}$ to $V_{4NP}$ (4 doses)<br>• $V_{5P}$ to $V_{5NP}$ (5 doses)<br>• $V_{6P}$ to $V_{6NP}$ (6 doses) | Years | 0.38<br>1<br>4<br>10<br>20<br>45 | [0.13, 0.88]<br>[0.08, 3]<br>[3, 5]<br>[5, 15]<br>[10, 30]<br>[30, 60] | Uniform | (Roper et al., 2018; World Health Organization, 2017, 2018) |
| $\tau_{mi}$ | Immunity waning of maternal antibodies in infants (average | Months | 6 | [3, 12] | Uniform | (Amirthalingam et al., 2014, 2016; Dabrera et al., 2015) |

| | | | | | | |
|---|---|---|---|---|---|---|
| | duration of protection in infants)~ | | | | | |
| $\tau_{mm}$ | Immunity waning of maternal antibodies in mothers (average duration of protection in mothers from ANC doses) for prior immunity level 1 (lowest) | Years | 5 | [3, 10] | Uniform | (World Health Organization, 2019) |
| $\varepsilon_1$<br>$\varepsilon_2$<br>$\varepsilon_3$<br>$\varepsilon_4$<br>$\varepsilon_5$<br>$\varepsilon_6$ | Vaccine effectiveness (population fully protected against infection)<br>• 1 dose<br>• 2 doses<br>• 3 doses<br>• 4 doses<br>• 5 doses<br>• 6 doses | % | 0<br>80<br>99<br>99<br>99<br>99 | -<br>[70, 90]<br>[98, 100]<br>[98, 100]<br>[98, 100]<br>[98, 100] | Uniform | (Roper et al., 2018; World Health Organization, 2017, 2018, 2019) |
| $\varepsilon_{m1}$<br>$\varepsilon_{m2}$<br>$\varepsilon_{m3}$ | Maternal antibody effectiveness (against maternal and neonatal tetanus) based on prior immunity:<br>• Lowest level of prior immunity<br>• Mid-level of prior immunity<br>• Highest level of prior immunity | % | 0<br>94<br>99 | -<br>[80, 98]<br>[98, 100] | Uniform | (Blencowe et al., 2010; Roper et al., 2007, 2018; World Health Organization, 2017, 2018, 2019) |

Notes: ^Time-related parameters are shown in natural units (e.g. days, months, years). These parameters are transformed to a rate (1/duration) when applied in the model. *Distribution for Latin hypercube sampling. ~Informed by studies on duration of maternal antibodies in infants following DTPCV vaccination based on studies of pertussis. Abbreviations: DTPCV, diphtheria, tetanus, pertussis-containing vaccine.

## 5. Technical Expert Group

The role of the technical expert group (TEG) was to advise on the model development, epidemiology and immunology of disease, and broader project guidance. The parameter values were established with input and review from the TEG.

- Dr Ampeire Immaculate, Ugandan National Immunization Program, Ministry of Health, Government of Uganda.
- Dr Anna Acosta, Meningitis and Vaccine Preventable Diseases Branch at U.S. Centers for Disease Control and Prevention (CDC)
- Dr Annet Kisakye, Expanded Program on Immunization, World Health Organization, Uganda
- Prof Paula Mendes Luz, Instituto Nacional de Infectologia Evandro Chagas (INI)
- Dr Todi Mengistu, Measurement, Evaluation and Learning (MEL) team at Gavi, the Vaccine Alliance
- Dr Helen Quinn, National Centre for Immunisation Research and Surveillance (NCIRS); University of Sydney Children's Hospital Westmead Clinical School.
- So Yoon Sim, Value of Vaccines, Modeling & Economics (VoV) team, Immunization Analysis & Insights (IAI) unit, Department of Immunization, Vaccines and Biologicals (IVB), World Health Organization
- Dr Rania Tohme, Hepatitis B and Tetanus Team in the Global Immunization Division at the U.S. Centers for Disease Control and Prevention (CDC)

**References**


Amirthalingam, G., Andrews, N., Campbell, H., Ribeiro, S., Kara, E., Donegan, K., Fry, N. K., Miller, E., & Ramsay, M. (2014). Effectiveness of maternal pertussis vaccination in England: An observational study. *The Lancet*, *384*(9953), 1521–1528. https://doi.org/10.1016/S0140-6736(14)60686-3

Amirthalingam, G., Campbell, H., Ribeiro, S., Fry, N. K., Ramsay, M., Miller, E., & Andrews, N. (2016). Sustained Effectiveness of the Maternal Pertussis Immunization Program in England 3 Years Following Introduction. *Clinical Infectious Diseases: An Official Publication of the Infectious Diseases Society of America*, *63*(Suppl 4), S236–S243. https://doi.org/10.1093/cid/ciw559

Blencowe, H., Lawn, J., Vandelaer, J., Roper, M., & Cousens, S. (2010). Tetanus toxoid immunization to reduce mortality from neonatal tetanus. *International Journal of Epidemiology*, *39*(Suppl 1), i102–i109. https://doi.org/10.1093/ije/dyq027

Dabrera, G., Amirthalingam, G., Andrews, N., Campbell, H., Ribeiro, S., Kara, E., Fry, N. K., & Ramsay, M. (2015). A Case-Control Study to Estimate the Effectiveness of Maternal Pertussis Vaccination in Protecting Newborn Infants in England and Wales, 2012–2013. *Clinical Infectious Diseases*, *60*(3), 333–337. https://doi.org/10.1093/cid/ciu821

Roper, M. H., Vandelaer, J. H., & Gasse, F. L. (2007). Maternal and neonatal tetanus. *The Lancet*, *370*(9603), 1947–1959. https://doi.org/10.1016/S0140-6736(07)61261-6

Roper, M. H., Wassilak, S. G., Scobie, H. M., Ridpath, A. D., & Orenstein, W. A. (2018). Tetanus toxoid. In *Plotkin's Vaccines* (7th ed, pp. 1052–1080). PA: Elsevier.

World Health Organization. (2017). *Tetanus vaccines: WHO Position Paper – February 2017* (No. Volume 92, 06; Weekly Epidemiological Record). World Health Organization. https://www.who.int/publications/i/item/WHO-WER9206

World Health Organization. (2018). *The Immunological Basis for Immunization Series Module 3: Tetanus*. World Health Organization. https://www.who.int/publications-detail-redirect/9789241513616

World Health Organization. (2019). Protecting all against tetanus: Guide to sustaining maternal and neonatal tetanus elimination (MNTE) and broadening tetanus protection for all populations. World Health Organization. https://iris.who.int/handle/10665/329882
